# Urodynamic evaluation of neurogenic lower urinary tract dysfunction in children with Guillain-Barre syndrome within 6 months

**DOI:** 10.3389/fneur.2025.1706698

**Published:** 2025-12-08

**Authors:** Yan Long, Hui Zhang, Naixi Zhang

**Affiliations:** 1 West China Second University Hospital, Sichuan University, Chengdu, Sichuan, China; 2 Sichuan Provincial Children’s Hospital, Meishan, Sichuan, China; 3 Key Laboratory of Birth Defects and Related Diseases of Women and Children (Sichuan University), Ministry of Education, Chengdu, Sichuan, China

**Keywords:** Guillain-Barre syndrome, neurogenic urinary bladder, urinary retention, urodynamics, children

## Abstract

**Objective:**

This study aimed to evaluate urodynamic characteristics, identify high-risk and unfavorable urodynamic findings in children with neurogenic lower urinary tract dysfunction (NLUTD) secondary to Guillain-Barre syndrome (GBS) within 6 months since the onset of illness.

**Methods:**

This study conducted a retrospective observational analysis of 16 children diagnosed with GBS complicated by NLUTD. Clinical data collected included demographic information, lower urinary tract symptoms, uroflowmetry results, post-void residual urine (PVRU), and urodynamic parameters. Follow-up assessments focused on high-risk urodynamic features-such as high detrusor pressure during the filling phase and low bladder compliance-as well as unfavorable findings, including detrusor overactivity, detrusor underactivity, or an acontractile bladder.

**Results:**

The majority of patients presented with clinical symptoms of urinary retention. Characterized by a decreased Qmax on uroflowmetry and elevated PVRU. Urodynamic study (UDS) revealed two predominant findings: reduced bladder sensation during the filling phase and detrusor underactivity during the voiding phase. At the 8-week follow-up, low bladder compliance and detrusor overactivity were observed in only 2 of the 16 patients (12.5%). During the filling phase, none of the patients exhibited high detrusor pressure (>40 cmH₂O), while all cases demonstrated underactive bladder function during the voiding phase. By the 24-week follow-up, 12 patients (12/16, 75.0%) had achieved normal bladder compliance, with only one case persisting with low bladder compliance and detrusor overactivity. Additionally, 10 patients (10/16, 62.5%) who had presented with an underactive bladder during the voiding phase showed recovery by the 24-week assessment.

**Conclusion:**

The prognosis of autonomic dysfunction involving the urinary system in pediatric GBS was generally favorable, with most cases of GBS-associated NLUTS showing substantial reversibility, as confirmed by the normalization of urodynamic parameters over time.

## 1 Introduction

Guillain-Barre syndrome (GBS) is an acute-onset immune-mediated, inflammatory disease of the peripheral nervous system (PNS) and is one of the common causes of acute flaccid paralysis (1). It is a monophasic illness that typically presents with relatively symmetrical weakness of the limbs, which rapidly progresses over time (1, 2). The annual incidence of the disease is reported to be 1–2/100,000 per year, with male predominance (1.5:1). Children are reported to be less affected (0.34–1.34 per 100,000 per year) than adults (3, 4).

In patients with GBS, autonomic dysfunction may involve the urinary system, characterized by neurogenic lower urinary tract dysfunction (NLUTD). Some patients exhibit urinary retention in the early stages, primarily due to transient autonomic dysregulation or somatic nerve involvement (4, 5). The detrusor muscle weakness results from impaired parasympathetic input (pelvic splanchnic nerves, S2-S4), while sympathetic overactivity (T11-L2) may increase bladder outlet resistance. In addition, inflammation affecting the pudendal nerve (S2-S4) can lead to external sphincter dyssynergia, further contributing to functional obstruction (5–7). Although urinary retention is typically transient and resolves with disease stabilization, temporary catheterization may be required to prevent complications (6, 7).

Urodynamic study (UDS) is the primary objective method for evaluating NLUTD, such as urinary retention, urinary incontinence (8, 9). Unfavorable urodynamic parameters showed in UDS are widely recognized as high-risk indicators for renal complications, and initiating proper management of NLUTD could mitigate the upper urinary tract complications in children (9, 10). This study aimed to evaluate urodynamic characteristics, detect unfavorable urodynamic findings of children with GBS presenting NLUTD and follow up these findings at different time intervals.

## 2 Materials and methods

We conducted a retrospective chart review of 16 children diagnosed with GBS who had complicated with NLUTD at our hospital from June 2021 to December 2024. A comprehensive medical history, physical examinations, cerebrospinal fluid (CSF), nerve conduction studies (NCS) consistent with polyneuropathy, were performed to ensure accurate diagnosis of GBS. All of patients showed clinical symptoms of lower urinary tract dysfunction, including urinary retention, urgency and/or urinary incontinence. Exclusion criteria included urinary tract infections, non-neurogenic bladder, structural bladder abnormalities (e.g., bladder cancer, bladder calculi), or psychogenic urinary frequency. Informed consent was obtained from all of the children’s parents. The study was conducted in accordance with the ethical principles of the Declaration of Helsinki, and was approved by the Independent Ethics Committee/Institutional Review Board of our hospital.

The clinical data collected encompassed demographic details, lower urinary tract symptoms, and a 2-day frequency volume chart. Additionally, each participant underwent uroflowmetry, post-voided residual urine (PVRU) measurement, and UDS. A standardized urodynamic detector (Laborie®) was utilized, with all procedures were conducted according to the standard methods recommended by the International Continence Society by the same physician, and results of UDS interpreted by experienced pediatric nephrologists who specifically trained in pediatric urodynamics and participated actively in UDS.

The outcome measures were evaluated through urodynamic parameters, including the results of filling cystometry (bladder capacity, bladder compliance, detrusor overactivity), the results of the voiding phase (maximum detrusor pressure during the voiding phase), and PVRU. Cystometric bladder capacity was defined as the bladder volume at the end of the filling cystogram. The expected bladder capacity (EBC) for children was calculated using the formula: EBC (ml) = [age (years) + 1] × 30. The EBC is applicable for children between 4 and 12 years as it reaches a level of 390 mL at 12 years. Based on this calculation, bladder capacity was classified as: “Increased”: if the measured maximum cystometric capacity was >150% of the EBC; Decreased: if the measured maximum cystometric capacity was <65% of the EBC (11). Bladder compliance was calculated by dividing the change in volume by the change in detrusor pressure during filling cystometry. The maximum detrusor pressure during the voiding phase and PVRU at the end of micturition were recorded.

High-risk findings (high detrusor pressure during the filling phase, low-compliance bladder) and unfavorable findings (detrusor overactivity, underactive or acontractile bladder) were observed and followed up within 6 months since the onset of illness. High detrusor pressure exceeding 40cmH_2_O during the filling phase was considered as a high-risk finding of urodynamic parameter to pose significant risk to the upper urinary tract (8, 11). Low bladder compliance was recognized as a compliance value lower than 20 mL/cmH_2_O, while high compliance was higher than 40 mL/cmH_2_O (11, 12). Detrusor overactivity was defined as involuntary detrusor contraction during the filling phase, with a pressure rise exceeding 15cmH_2_O above baseline (8, 11, 12). During the voiding phase, detrusor function was classified into three categories: normal, underactive, or acontractile bladder. An underactive bladder was defined as reduced detrusor pressure (<40cmH₂O), leading to prolonged bladder emptying or the inability to achieve complete emptying. An acontractile bladder was characterized by the absence of any increase in detrusor pressure during voluntary voiding (11).

All the pediatric participants and their parents received personalized lifestyle recommendations, focusing on balanced fluid intake and dietary adjustments. Furthermore, specialists provided guidance on clean intermittent catheterization (CIC) and behavioral modifications to improve voiding habits. Besides, 16 patients received biofeedback lasting 30 min each time, administered twice weekly using a specialized apparatus (Laborie®). During the procedure, children were positioned laterally, with a myoelectric probe inserted into the anus and surface electromyography electrodes attached to the perianal skin. They were then guided by multimedia animations displayed on a computer screen to perform pelvic floor muscle contractions and relaxations, ensuring proper and effective exercise.

Continuous variables with a normal distribution were presented as mean ± standard deviation, while continuous variables with a skewed distribution were presented as median and interquartile range. Categorical variables were summarized as frequencies and percentages. Statistical analyses were performed with SPSS software (version 18.0). Clinical variables were compared across groups using the Pearson chi-square test for categorical data. All reported *p* values <0.05 were considered statistically significant.

## 3 Results

### 3.1 Baseline characteristics of the study cohort

Sixteen participants (9 males and 7 females), with a median age of 10.5 ± 2.2 years, met the inclusion criteria. Detailed clinical features, including age, sex, lower urinary tract symptoms, uroflowmetry results and urodynamic parameters were summarized in Table 1.

**Table 1:** Clinical characteristics of 16 children diagnosed with GBS who had complicated with NLUTD.

Patient No.	Age (years)	Sex	Lower urinary tract symptoms			Uroflowmetry	Filling cystometry			Voiding phase	PVRU (ml)
			Urinary retention	Urgency	Urinary incontinence	Qmax (ml/min)	Bladder capacity (ml)	Bladder compliance (ml/cmH₂O)	Detrusor overactive	Maximum detrusor pressure (mmH_2_O)	
1	11.7	Male	Yes	No	No	9.5	470	23	No	16	230
2	9.6	Male	Yes	No	No	10.3	410	45	No	10	190
3	10.2	Female	Yes	No	Yes	9.7	500	46	No	12	220
4	9.4	Male	Yes	No	No	8.4	460	42	No	15	180
5	8.3	Female	Yes	No	No	7.3	350	34	No	13	170
6	12.6	Male	Yes	No	No	10.2	560	27	No	23	270
7	12.7	Male	Yes	No	No	9.8	590	46	No	15	160
8	8.7	Female	Yes	No	No	6.3	350	25	No	16	170
9	9.5	Female	No	Yes	Yes	6.9	150	10	Yes	24	80
10	10.5	Male	Yes	No	No	9.1	430	22	No	18	220
11	11.4	Female	Yes	No	Yes	10.2	540	48	No	14	210
12	8.4	Male	Yes	No	No	5.8	410	45	No	12	150
13	9.0	Female	Yes	No	No	6.0	370	21	No	17	230
14	12.2	Female	No	Yes	Yes	11.7	190	9	Yes	28	70
15	11.8	Male	Yes	No	No	6.3	470	21	No	19	240
16	10.9	Male	Yes	No	No	7.1	490	44	No	18	160

GBS, Guillain Barre syndrome; NLUTD, Neurogenic lower urinary tract dysfunction; Qmax, Maximum flow rate; PVRU, Post-voided residual urine.

Fourteen patients (14/16, 87.5%) presented with clinical symptoms of urinary retention, while two cases (2/16, 12.5%) were complicated by urinary incontinence. All patients exhibited a decreased maximum flow rate (Qmax) accompanied by an increase of PVRU. Additionally, UDS revealed a reduction in the maximum detrusor pressure during the filling phase, and abdominal straining during micturition observed. Among them, two cases (2/16, 12.5%) demonstrated clinical manifestations of urinary urgency with urge incontinence. Urodynamic evaluation in these two patients (2/16, 12.5%) indicated a decreased functional bladder capacity and the presence of detrusor overactivity during the filling phase.

### 3.2 Results of high-risk and unfavorable urodynamic findings in participants at follow-ups

Table 2 summarized the high-risk and unfavorable urodynamic findings of 16 patients within 6 months since the onset of illness.

**Table 2:** Results of high-risk and unfavorable urodynamic findings in participants at follow-ups.

Patients No.	Onset of illness				At 8 weeks				At 24 weeks			
	Bladder compliance (ml/cmH₂O)	High detrusor pressure (>40 cmH_2_O) at the filling phase	Detrusor overactive	Detrusor function at voiding	Bladder compliance (ml/cmH₂O)	High detrusor pressure (>40 cmH2O) at the filling phase	Detrusor overactive	Detrusor function at voiding	Bladder compliance (ml/cmH₂O)	High detrusor pressure (>40 cmH2O) at the filling phase	Detrusor overactive	Detrusor function at voiding
1	23	No	No	Underactive	26	No	No	Underactive	28	No	No	Normal
2	45	No	No	Underactive	43	No	No	Underactive	35	No	No	Normal
3	46	No	No	Underactive	43	No	No	Underactive	42	No	No	Underactive
4	42	No	No	Underactive	35	No	No	Underactive	32	No	No	Normal
5	34	No	No	Underactive	32	No	No	Underactive	34	No	No	Normal
6	27	No	No	Underactive	28	No	No	Underactive	29	No	No	Normal
7	46	No	No	Underactive	44	No	No	Underactive	42	No	No	Underactive
8	25	No	No	Underactive	27	No	No	Underactive	28	No	No	Normal
9	10	No	Yes	Underactive	14	No	Yes	Underactive	21	No	No	Normal
10	22	No	No	Underactive	26	No	No	Underactive	29	No	No	Normal
11	48	No	No	Underactive	43	No	No	Underactive	36	No	No	Underactive
12	45	No	No	Underactive	42	No	No	Underactive	43	No	No	Underactive
13	21	No	No	Underactive	26	No	No	Underactive	28	No	No	Normal
14	9	No	Yes	Underactive	13	No	Yes	Underactive	16	No	Yes	Underactive
15	21	No	No	Underactive	27 L	No	No	Underactive	26	No	No	Normal
16	44	No	No	Underactive	42	No	No	Underactive	34	No	No	Underactive

At the 8-week, low bladder compliance and detrusor overactivity were observed in only 2 of the 16 patients (12.5%). None of the patients exhibited high detrusor pressure (>40 cmH₂O) during the filling phase, while all cases demonstrated underactive bladder function during the voiding phase.

By the 24-week follow-up, 12 patients (12/16, 75.0%) had achieved normal bladder compliance, with only one case (1/16, 6.3%) persisting with low bladder compliance and detrusor overactivity. Similarly, no patient showed detrusor pressure exceeding 40cmH₂O during the filling phase. Additionally, 10 patients (10/16, 62.5%) who had presented with an underactive bladder during the voiding phase showed recovery by the 24-week assessment.

## 4 Discussion

GBS, as an immune-mediated, rapidly progressive polyneuropathy, can be classified into several subtypes based on clinical manifestations and electrophysiological characteristics (3). During the peak phase of the illness in pediatric populations, approximately 75% of patients lose the ability to walk independently, 30% develop tetraparesis, 35–50% exhibit cranial nerve involvement, and 15–20% experience respiratory failure and/or autonomic dysfunction (3, 4). Although GBS primarily affects motor and sensory nerves, autonomic dysfunction-though less common-can lead to NLUTD, significantly affecting the patient’s quality of life (5–7).

The uroflowmetry is a non-invasive procedure and the first-line method for evaluating the function and dysfunction of the lower urinary tract (9). It involves measuring parameters such as flow rate, voided volume, voiding time, and analyzing the uroflow pattern (9–11). Based on our study, all patients exhibited a decreased Qmax along with elevated PVRU. Their flow curves consistently demonstrated an interrupted pattern, indicative of underactive bladder. Each peak in the flow curve corresponded to abdominal straining, which served as the primary driving force for urination. Flow ceased between successive straining episodes.

UDS is not routinely used to evaluate lower urinary tract function in neurologically intact children, but is regularly performed in those with suspected neurogenic bladder (NB) (7, 9). This study assesses both the filling and voiding phases of bladder function. Cystometry, in particular, refers to the urodynamic investigation conducted during the filling phase of the micturition cycle. Bladder storage function should be characterized using objective parameters, including bladder capacity, bladder compliance, and detrusor activity (11, 12). In our cohort, the bladder capacity of vast majority of patients (14/16, 87.5%) increased accompanied by reduced bladder sensation during the filling phase; only two cases (2/16, 12.5%) showed decreased bladder capacity with detrusor overactivity. Pressure-volume relationship was evaluated during the voiding phase, and an underactive bladder was the predominant finding of NLUTD during the acute phase in all 16 patients. In our study of urodynamic evaluation, high-risk and unfavorable urodynamic findings of 16 patients-such as low bladder compliance and detrusor overactivity-were observed in only two patients (2/16, 12.5%) within the 6-month period. After 6 months of follow-up, urodynamic parameters normalized in the majority of patients, an outcome facilitated in part by biofeedback therapy. Specifically, 10 patients (10/16, 62.5%) who had presented with an underactive bladder during the voiding phase showed recovery by the 24-week assessment. These results indicated that the prognosis of NLUTS for children with GBS was likely to generally more favorable (13–15).

However, this study had several limitations. First, the retrospective chart review design inherently limited the availability and consistency of data. Second, the potential influence of biofeedback therapy on the urodynamic parameters could not be definitively assessed, such as maximal cystometric capacity, bladder compliance, and detrusor pressure in the context of neurogenic bladder. Third, the small sample size may restrict the generalizability of the findings. Future prospective studies incorporating larger cohorts and longitudinal repeated urodynamic evaluations are warranted to validate these results and clarify the long-term outcomes.

## 5 Conclusion

In conclusion, NLUTD secondary to GBS in children primarily manifested as urinary retention, characterized by a decreased Qmax on uroflowmetry and elevated PVRU. UDS revealed two predominant findings: reduced bladder sensation during the filling phase and detrusor underactivity during the voiding phase. The prognosis for pediatric GBS patients was generally favorable, with most cases of GBS-associated NLUTS showing substantial reversibility, as confirmed by the normalization of urodynamic parameters over time.

## Data Availability

The original contributions presented in the study are included in the article/supplementary material, further inquiries can be directed to the corresponding author.
